# In Response

**DOI:** 10.4269/ajtmh.16-0130b

**Published:** 2016-07-06

**Authors:** Nancy Fullman, Felix Masiye, Emmanuela Gakidou

**Affiliations:** ^1^ Institute for Health Metrics and Evaluation, University of Washington, Seattle, Washington. E-mail: nf4@uw.edu.; ^2^ School of Humanities and Social Sciences, University of Zambia, Lusaka, Zambia. Email: fmasiye@yahoo.com.; ^3^ Institute for Health Metrics and Evaluation, University of Washington, Seattle, Washington. Email: gakidou@uw.edu.

Dear Sir:

Zambia has achieved marked progress in improving child survival, with national levels of under-five mortality falling to 37% from 1990 to 2010.^1^ Recent analyses found an accelerated reduction in under-five mortality since 2000,^2^ which was attributed to Zambia's simultaneous scale-up of four interventions: malaria vector control, the pentavalent vaccine, exclusive breastfeeding, and prevention of mother-to-child transmission of human immunodeficiency virus (HIV). These results stress the importance of taking a holistic view of child health priorities in Zambia, ranging from disease burden to the delivery of life-saving interventions, across local contexts.

To determine which health programs and policies to prioritize, decision-makers require several types of information, such as national levels and trends in mortality and disease burden data. Results from the Global Burden of Disease Study show that malaria was the leading cause of total under-five deaths, as well as under-5 deaths per 100,000, in Zambia in 2000 and 2010).^3^,^4^ Four other causes—diarrheal diseases, lower respiratory infections, HIV, and protein–energy malnutrition—were consistently among the leading five killers of Zambian children during this time. Yet statistics on mortality and disease burden on their own cannot fully answer the questions of why certain trends occur and what countries like Zambia should do to further advance the health of its children.

Decision-makers also need evidence on the access to and use of key health interventions, as well as how well interventions work to improve health outcomes. Without this information, it is quite challenging to optimally prioritize health program funding and implementation. For years, many regarded Zambia's scale-up of malaria vector control as the primary impetus for reductions in under-five mortality.^5^–^7^ We found that Zambia scaled up several interventions, including malaria vector control, between 2000 and 2010,^2^,^8^ which collectively contributed to Zambia's heightened pace of decreasing under-five mortality. Rates exclusive breast-feeding, for example, rapidly rose from an average of 38% in 2000 to 80% in 2010,^8^ likely reflecting Zambia's investments in tackling child malnutrition, particularly among infants.^9^

With our study, we provide a novel evaluation of how trends in under-five mortality were related to a broad range of interventions, including malaria vector control, and other determinants of health. Although we could not include every possible driver of improved child survival, our analyses offer a robust framework for future—and expanded—assessments of child health priorities in Zambia. We encourage researchers and decision-makers alike to view priority setting as a multifaceted process that requires a full spectrum of information, including, but not limited to, measures of disease burden. To make the greatest impact, we must draw from evidence that reflects the complex interplay of burden, interventions, and other drivers—and how, collectively, this information can be used to most effectively improve child health.
